# Entomological Surveillance of the Invasive *Aedes* Species at Higher-Priority Entry Points in Northern Iran: Exploratory Report on a Field Study

**DOI:** 10.2196/38647

**Published:** 2022-10-31

**Authors:** Seyed Hassan Nikookar, Alireza Maleki, Mahmoud Fazeli-Dinan, Razieh Shabani Kordshouli, Ahmadali Enayati

**Affiliations:** 1 Department of Medical Entomology and Vector Control Health Sciences Research Center, School of Public Health Mazandaran University of Medical Sciences Sari Iran; 2 Guilan University of Medical Sciences Rasht Iran; 3 Department of Medical Entomology and Vector Control School of Public Health and Health Sciences Research Center Mazandaran University of Medical Sciences Sari Iran

**Keywords:** mosquito surveillance, *Aedes*, biodiversity, Guilan, Northern Iran

## Abstract

**Background:**

Arboviral diseases such as dengue, Zika, and chikungunya are transmitted by *Aedes aegypti* and *Ae albopictus* and are emerging global public health concerns.

**Objective:**

This study aimed to provide up-to-date data on the occurrence of the invasive *Aedes* species in a given area as this is essential for planning and implementing timely control strategies.

**Methods:**

Entomological surveillance was planned and carried out monthly from May 2018 to December 2019 at higher-priority entry points in Guilan Province, Northern Iran, using ovitraps, larval collection, and human-baited traps. Species richness (*R*), Simpson (*D*), evenness (*E*), and Shannon-Wiener indexes (*H̕*) were measured to better understand the diversity of the *Aedes* species. The Spearman correlation coefficient and regression models were used for data analysis.

**Results:**

We collected a total of 3964 mosquito samples including 17.20% (682/3964) belonging to the *Aedes* species, from 3 genera and 13 species, and morphologically identified them from May 2018 to December 2019. *Ae vexans* and *Ae geniculatus*, which showed a peak in activity levels and population in October (226/564, 40.07% and 26/103, 25.2%), were the eudominant species (*D*=75.7%; *D*=21.2%) with constant (C=100) and frequent (C=66.7%) distributions, respectively. The population of *Ae vexans* had a significant positive correlation with precipitation (*r*=0.521; *P*=.009) and relative humidity (*r*=0.510; *P*=.01), whereas it was inversely associated with temperature (*r*=−0.432; *P*=.04). The Shannon-Wiener Index was up to 0.84 and 1.04 in the city of Rasht and in July, respectively. The rarefaction curve showed sufficiency in sampling efforts by reaching the asymptotic line at all spatial and temporal scales, except in Rasht and in October.

**Conclusions:**

Although no specimens of the *Ae aegypti* and *Ae albopictus* species were collected, this surveillance provides a better understanding of the native *Aedes* species in the northern regions of Iran. These data will assist the health system in future arbovirus research, and in the implementation of effective vector control and prevention strategies, should *Ae aegypti* and *Ae albopictus* be found in the province.

## Introduction

### Background

Mosquitoes are the most important medical insects because they transmit various pathogens to humans and animals [1]. Mosquitoes are found in temperate and tropical regions of the world and beyond the Arctic Circle. The family *Culicidae* includes 3591 valid species, which are classified into 2 subfamilies and 113 genera [2]. The genera *Anopheles*, *Culex*, and *Aedes* are the most important taxa in this family. The genus *Aedes* has the highest number of species, with 33 species of uncertain subgeneric status and 900 species classified into 72 subgenera [1]. Members of the genus *Aedes* are vectors of at least 22 arboviruses, including some of the most human health–threatening viruses such as the chikungunya, dengue, and Zika viruses [3].

*Aedes aegypti* and *Ae albopictus* are the main vectors of these arboviral diseases. *Ae aegypti* is a domestic species with highly synanthropic behavior, originating from the forests of Africa, and is currently found in most tropical and subtropical regions around the world [4,5]. *Ae albopictus* is native to the forests of Southeast Asia that subsequently spread to the Americas, Europe, Africa, Australia, and several islands in the Pacific Ocean over the past 30 to 40 years, following global trade, especially in used tires [6,7].

Over the past 2 decades, the Asian tiger mosquito, *Ae albopictus*, and yellow fever mosquito, *Ae aegypti*, have been reported in several countries of the Mediterranean basin, including Afghanistan, Armenia, Oman, Pakistan, Saudi Arabia, Yemen, and Turkey (which is located near the Mediterranean basin) [8,9]. Dengue and chikungunya outbreaks have recently been reported in Pakistan, Saudi Arabia, Yemen, and Oman [8-10], raising concerns about the probable influx of these species in Iran [11]. As anticipated, in 2014, the first specimens of *Ae albopictus* were collected from Sistan and Baluchestan Province in neighboring Pakistan [12]. More importantly, *Ae aegypti* has also been revealed in the Lengeh and Khamir ports in Hormozgān Province during the last couple of years [9], which has caused great concern for the country. This may result in outbreaks of arboviral diseases in Iran, where *Ae aegypti* and *Ae albopictus* are established. Therefore, it strongly emphasizes the necessity for regular implementation of entomological surveillance programs at points of entry to detect the presence of invasive *Aedes* species and to estimate the risk of incidence of vector-borne diseases throughout the county, especially in Guilan Province.

Over the past few decades, Guilan Province has made tremendous efforts in social development and urbanization, the expansion of agricultural projects, water resources, and the tourism industry. There are several ports of entry, including the Anzali and Astara international ports, which link it to Eurasia through the Volga Don Canal [13], and Rasht Sardar Jangal International Airport in the province. Suitable weather conditions and a spectacular natural landscape make the province an important national and international holiday destination in Northern Iran, factors that predispose the province to the risk of invasive *Aedes* species.

### Objectives

Therefore, given the concern about the possible entry of these species from neighboring northern countries as well as the geographical and ecological suitability of Guilan Province, this study aims to conduct and establish an initial entomological surveillance of invasive species of *Aedes* in line with the national search for *Ae aegypti* and *Ae albopictus.* In doing so, apart from early detection of the entry of these invasive species, capacity building of the workforce of the Health Deputy and other organizations such as seaports and airports of Guilan Province, as well as providing a data set for the fauna of *Aedes* in Northern Iran were among the purposes of this research.

As no biodiversity studies on *Aedes* species in Guilan Province, Northern Iran, have been conducted so far, assessment of the biodiversity of *Aedes* mosquitoes is one of the aims of this study.

## Methods

### Study Area

Guilan Province is located in northwestern Iran between 36°34′ and 38°27′ N latitude and 48°34′ and 50°36′ E longitude and has mostly coastal, plain, foothill, and mountainous areas with a population of 2,531,000 and an area of approximately 14,042 km^²^ The province is surrounded by the Republic of Azerbaijan and the Caspian Sea to the north, Ardebil Province to the west, Mazandaran Province to the east, and Zanjan Province to the south. The center of the province, Rasht City, is known internationally as the “City of Silver Rains” and among Iranians as the “City of Rain.” The maximum and minimum absolute temperatures are 37 °C and −19 °C, respectively, and the average temperature is 15 °C (30-year data from Guilan Synoptic Station) [14]. The average relative humidity at 06:30 AM was 94%, and at noon it was 72%. The average annual rainfall is approximately 1401 mm [15]. A moderate climate and abundance of water have turned the province into an ideal place for mosquitoes to thrive. Rice is a major crop in this province. The city of Astara in the far western part of Guilan Province is the most active transit port and the third most active border of the country between Iran and the Caucasus region, and is thus in a position that can support the entry of invasive *Aedes* into the country.

### Specimen and Data Collection

This study was started in 2017 in Guilan Province because of its strategic importance in the region in terms of the entry of invasive species into the country from the northern belt, by training the field work teams, organizing the study, and preparing the materials. The actual sampling was carried out bimonthly from May 2018 to December 2019, according to the seasonal activity of the species in the region. The specimens were identified to the species level, and the results were analyzed in 2020; this was followed by the drafting of the manuscript. Sampling was performed in 3 cities that harbored the main entry points, namely the Rasht International Airport and the Anzali and Astara seaports in Guilan Province. In each of these cities, the main entry point and 2 other locations in the vicinity were included in the sampling. This totals the sampling locations in each city (Rasht, Anzali, and Astara) to 3 locations (Multimedia Appendix 1), as suggested by the Iran Centers for Disease Control and Prevention surveillance guidelines for invasive *Aedes* vectors [9]. The specimens were collected using three methods: ovitrap, larval collection, and human-baited trap. A total of 27 staff members from the Guilan health centers were recruited and trained to perform sampling.

### Ovitrap Surveillance

**Figure 1:**
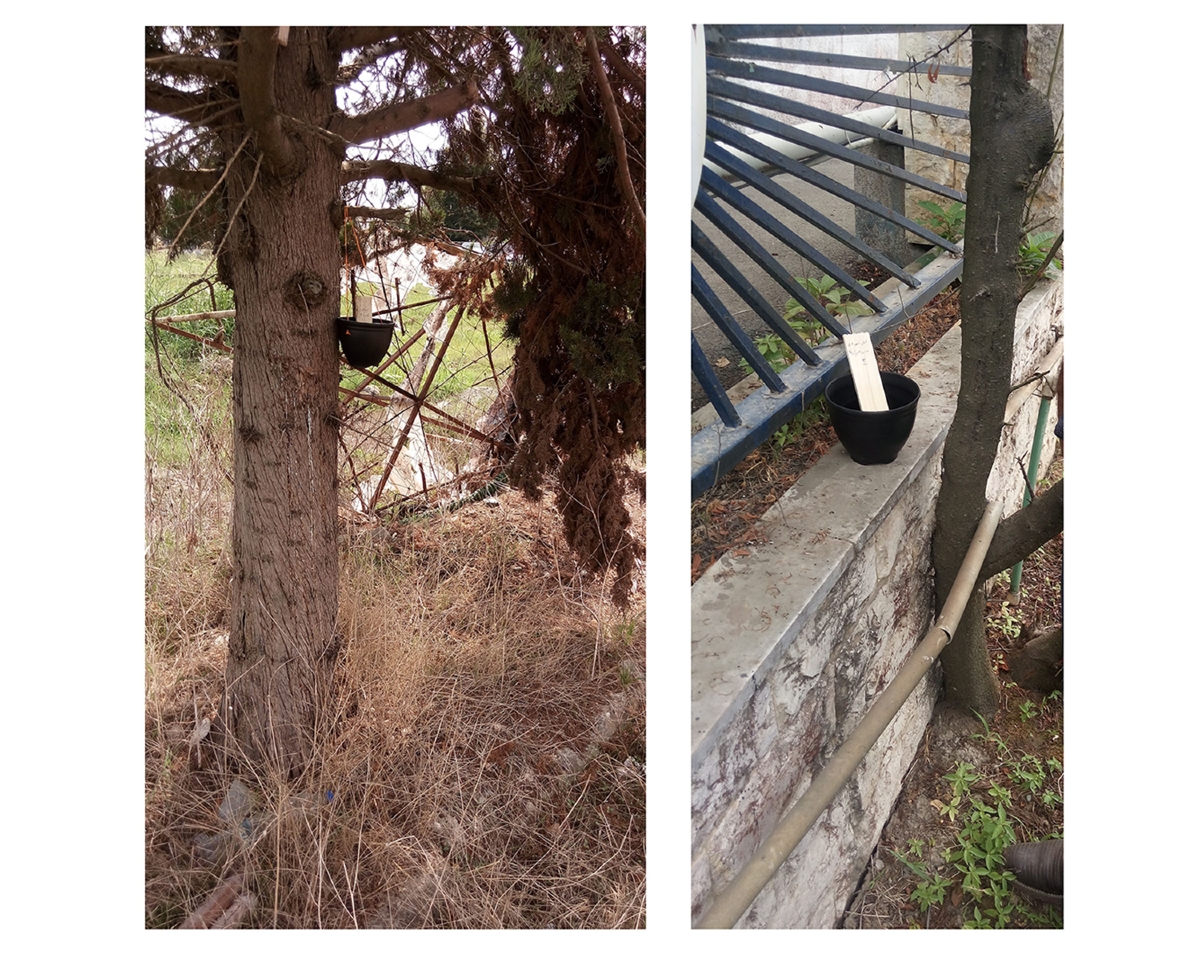
Ovitrap used to collect eggs of the invasive *Aedes* species (original photo).

The ovitraps were black 1 L plastic cylindrical buckets (12 cm in diameter × 15 cm in height) and wooden paddles (3 cm × 12 cm × 0.5 cm each) placed vertically inside the trap as a substrate for oviposition. A 10% solution of *Orayza sativa* or *Cynodon dactylon* was used as a natural attractant in the ovitraps. They were placed bimonthly (once in the first half of the month and once in the second half of the month) outdoors and indoors at a height of <1.5 m, protected from rain and direct sunlight, out of reach of children and pets at selected points at each point of entry (100 ovitraps in total), and visited 72 hours later. The suspected paddles were collected and transferred to the laboratory for counting and species identification after being kept for 2 to 3 days at room temperature before hatching (Figure 1). It should be mentioned that the ovitraps were mostly placed in roofed areas such as corners of buildings and roofed parking lots and inside buildings to protect them from rain, but for ovitraps that were placed in roofless environments, small gable roofs at a height of 30 cm were set up above each ovitrap. If this was not possible and rainwater entered the ovitrap, the excess water was automatically removed through a hole placed at the one-third mark from the upper end of the ovitrap.

### Larval Surveillance

Larval surveys were performed in the preferred natural and artificial habitats of invasive *Aedes* species using a standard 350 mL dipper. In small breeding sites where dippers could not be used, the sampling was performed using plastic pipettes. Sampling was always conducted by the same individual in the morning (from 8 AM to noon) or afternoon (from 4 PM to 6 PM) for approximately 30 minutes at each larval habitat. Approximately 10 to 30 dips were performed in each larval habitat, depending on their size. The collected larvae were preserved in glass vials containing lactophenol solution and transferred to the laboratory for being mounted on microscopic slides using Berlese medium before morphological identification.

### Human-Baited Catches

Daily biting was done each fortnight near breeding sites and human dwellings at each station (Multimedia Appendix 1) in the study cities. A total of 6 participants were randomly divided into 3 groups (each consisting of a human bait and a collector) for the 3 sampling stations in each city. The study was performed from 7 AM to 8 PM, with a break of 15 minutes every 3 hours. To avoid unnecessary biting, the participants were covered in net jackets in areas where the landing of mosquitoes was not taking place. The participants laid down and exposed their legs and hands from knee to ankle and elbow to wrist. The collector aspirated any mosquito that landed and gently expelled it into a paper cup covered with netting. The mosquitoes collected each hour were killed by freezing for at least 15 minutes at −20 °C, pinned, and identified using recent morphological keys [16]. It should be noted that before starting the collection, the aim of the research was explained to the participants and they were included in the study after providing informed consent.

### Ethics Approval

The study protocol was approved by the ethical committee of the Mazandaran University of Medical Sciences (IR.MAZUMS.REC.1397.3475) and the study was performed according to the Iran Centers for Disease Control and Prevention surveillance guidelines for invasive *Aedes* vectors [9].

### Dominance and Distribution of the *Aedes* Species

Dominance (D) and distribution (C) structures were also calculated for each of the species in the area according to the method proposed by Nikookar et al [17]. According to the obtained D and C values, 5 classes were considered to show the intensity of distribution and dominance.

### Biodiversity and Rarefaction Analysis

Indices of species richness, evenness, dominance, community heterogeneity, and sufficiency of sampling efforts were computed using the following formulas at the spatial and temporal scales:

Margalef (D_Mg_=S-1lnN), Menhinick (D_Mn_=SN), Simpson dominance (D=λ=i=1SPi2)), evenness (J or E or Pielou index) – (J=H’H’max=H’logS), Shannon indices (H=Σp_i_×lnp_i_) and rarefaction curve (E(Sn)=i=1S[1-N-NinNn]), where N represents the total number of individuals in the sample, S represents the number of species in the sample, N_i_ is the number of individuals of species number i; P_i_=niN, where P_i_ is the proportion of individuals observed in the ith species, n_i_ is the number of individuals in the ith taxon, and H′ is the Shannon-Wiener function [18-20].

It should be mentioned that the steep slope to the left of the curve indicates that many species have not yet been discovered, whereas reaching the asymptotic line indicates a reasonable number of specimens. Therefore, more intensive sampling efforts are likely to result in only a small number of additional species [20].

### Statistical Analysis

All statistical analyses were performed using SPSS (version 20, IBM Corp). The normality of the data was tested using the Shapiro-Wilk test, as the data were not normally distributed. Spearman correlation analysis was used to evaluate the relationship between the frequency of occurrence of *Aedes* species and climatic variables in the region. A regression model was also used to show the transparency and intensity of the relationship by using an *R*^2^ estimate.

## Results

### Species Composition

A total of 3964 mosquito specimens, including 2103 (53.05%) larvae and 1861 (46.94%) adults belonging to 3 genera, with 4 species being larvae, and 13 species being adults, were collected from Guilan Province, Northern Iran, from May 2017 to December 2017. Of these, 1.81% (38/2103) larvae and 21.82% (406/1861) adults belonged to the subfamiliy Anophelinae, and 98.19% (2065/2103) larvae and 78.18% (1455/1861) adults were from the subfamily Culicine (Table 1).

The highest number and percentage of samples were collected in Anzali (1412/3964, 35.62% of the total captured specimens), whereas the lowest number was collected in Astara (1158/3964, 29.21%). No *Anopheles* larvae were found in the studied counties, except for *Anopheles* *plumbeus* (Table 1).

*Ae vexans* was the most abundant species collected from all counties. *Ae vexans* was caught with maximum and minimum relative abundances in Anzali (256/1412, 18.13%) and Rasht (132/1394, 9.50%; Table 1). *Ae geniculatus*, *Ae echinus*, and *Ae pulchritarsis* were only collected as adults, with the former being the second most abundant *Aedes* species in this study.

**Table 1:** Numbers and percentage of mosquito species recorded at higher-priority entry points of Guilan Province, Northern Iran, from May 2018 to December 2019.

Species	Rasht			Anzali			Astara			Total	
	Larvae, n (%)	Adult, n (%)	Larvae, n (%)		Adult, n (%)	Larvae, n (%)		Adult, n (%)	Larvae, N (%)		Adult, N (%)
*Anopheles maculipennis sl*	—^a^	11 (1.8)	—		18 (2.5)	—		19 (3.5)	—		48 (2.6)
*Anopheles pseudopictus*	—	136 (22.3)	—		172 (24.2)	—		36 (6.6)	—		344 (18.5)
*Anopheles hyrcanus*	—	5 (0.8)	—		—	—		—	—		5 (0.5)
*Anopheles sacharovi*	—	—	—		—	—		9 (1.7)	—		9 (0.3)
*Anopheles plumbeus*	38 (4.8)	—	—		—	—		—	38 (1.8)		—
*Culex pipiens*	579 (74)	132 (21.6)	595 (84.8)		124 (17.5)	511 (82.7)		166 (30.5)	1685 (80.1)		417 (22.4)
*Culex theileri*	—	7 (1.1)	—		—	—		179 (32.8)	—		186 (10)
*Culex tritaeniorhynchus*	140 (18)	140 (23)	14 (2)		182 (25.6)	25 (4)		42 (7.7)	179 (8.5)		364 (19.6)
*Culex torrentium*	1 (0.1)	—	3 (0.4)		3 0.4()	—		—	4 (0.2)		3 (0.2)
*Aedes vexans*	25 (3.1)	107 (17.5)	90 (12.8)		166 (23.4)	82 (13.3)		94 (17.2)	197 (9.4)		367 (19.7)
*Aedes geniculatus*	—	58 (9.5)	—		45 (6.4)	—		—	—		103 (5.5)
*Aedes echinus*	—	14 (2.3)	—		—	—		—	—		14 (0.7)
*Aedes pulchritarsis*	—	1 (0.1)	—		—	—		—	—		1 0.05
Total	783 (100)	611 (100)	702 (100)		710 (100)	618 (100)		540 (100)	2103 (100)		1861 (100)

^a^No sample was collected.

### Monthly Population Trends of the *Aedes* Species

**Figure 2:**
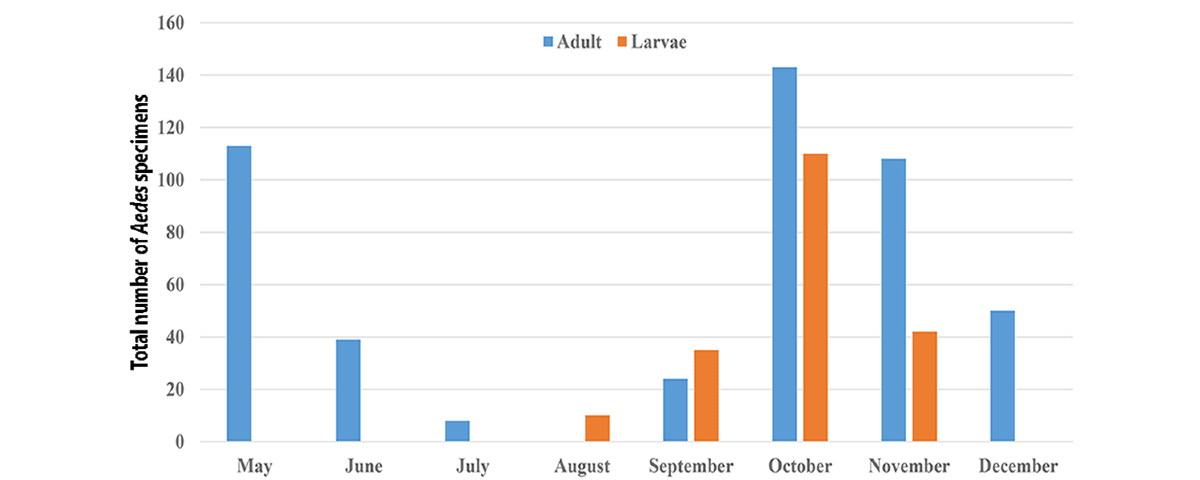
The total number of *Aedes* specimens collected by month at higher-priority entry points of Guilan Province, Northern Iran, from May 2018 to December 2019.

The highest total number of larvae (n=110) and adults of the (n=143) *Aedes* species was found in October, while the lowest number was found in August for larvae (10/197, 5.07%) and July for adults (8/485, 1.6%; Figure 2). The population density of *Ae vexans* adults in Rasht and Astara counties began to increase in May, disappeared from sampling in August, reached its greatest peak in October, and then gradually decreased. In Anzali, the species had a different population trend, appearing with 2 peaks, one in early May (48/55, 87%) and another at the beginning of autumn (45/59, 76%; Table 2). The highest number and percentage of *Ae vexans* larvae were recorded in October in Rasht (15/110, 13.6%) and Anzali (70/110, 63.6%) and in November in Astara (32/110, 76.2%; Table 3).

The *Ae geniculatus* species was active in all months except for August in Rasht and July and August in Anzali, although the species was not observed during the monthly sampling efforts in Astara. The highest population of the species was found in May in Rasht (17/42, 40%). After May, the population of the species decreased gradually in June and July, disappeared in August, and then increased and reached a smaller peak in October. The population of this species showed its highest peak in June (14/28, 50%) and October (14/59, 24%) in Anzali (Table 2). The population fluctuations of other species are shown in Table 2.

**Figure 3:**
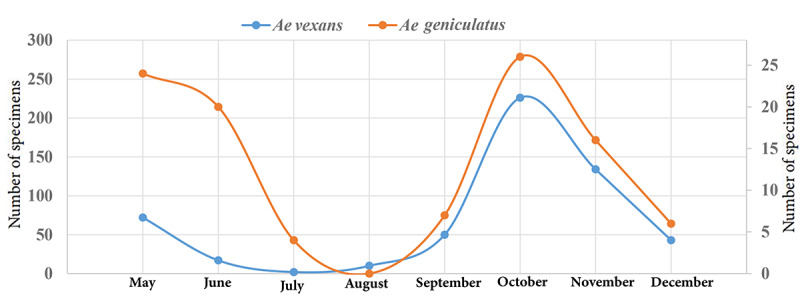
Monthly population trends of the most abundant species, *Aedes vexans* and *Ae geniculatus*, at higher-priority entry points of Guilan Province, Northern Iran, from May 2018 to December 2019.

The fluctuations that occurred between May and December in the populations of *Ae vexans* and *Ae geniculatus*, the most abundant species in the province, are shown in Figure 3.

**Table 2:** Monthly population fluctuations of adult *Aedes* species collected at higher-priority entry points of Guilan Province, Northern Iran, by collection month, 2018 to 2019.

County and species		May, n (%)	June, n (%)	July, n(%)	August, n (%)	September, n (%)	October, n (%)	November, n (%)	December, n (%)
**Rasht**									
	*Aedes vexans*	18 (42.8)	3 (27.3)	2 (25)	—^a^	4 (36.4)	35 (72.9)	30 (75)	15 (75)
	*Aedes geniculatus*	17 (40.5)	6 (54.5)	4 (50)	—	5 (45.4)	12 (25)	10 (25)	4 (20)
	*Aedes echinus*	7 (16.7)	2 (18.2)	2 (25)	—	2 (18.2)	—	—	1 (5)
	*Aedes pulchritarsis*	—	—	—	—	—	1 (2.1)	—	—
	Total	42 (100)	11 (100)	8 (100)	—	11 (100)	48 (100)	40 (100)	20 (100)
**Anzali**									
	*Aedes vexans*	48 (87.3)	14 (50)	—	—	6 (75)	45 (76.3)	33 (84.6)	20 (90.9)
	*Aedes geniculatus*	7 (12.7)	14 (50)	—	—	2 (25)	14 (23.7)	6 (15.4)	2 (9.1)
	Total	55 (100)	28 (100)	—	—	8 (100)	59 (100)	39 (100)	22 (100)
**Astara**									
	*Aedes vexans*	16 (100)	—	—	—	5 (100)	36 (100)	29 (100)	8 (100)
	Total	113 (23.3)	39 (8.04)	8 (1.64)	—	24 (4.95)	143 (29.48)	108 (22.26)	50 (10.30)

^a^No sample was collected.

**Table 3:** Monthly population fluctuations of larvae *Aedes* species collected at higher-priority entry points of Guilan Province, Northern Iran, by collection month, 2018 to 2019.

County	Species	May, n (%)	June, n (%)	July, n (%)	August, n (%)	September, n (%)	October, n (%)	November, n (%)	December, n (%)
Rasht	*Aedes vexans*	—^a^	—	—	10 (100)	—	15 (13.6)	—	—
Anzali	*Aedes vexans*	—	—	—	—	10 (28.57)	70 (63.6)	10 (23.8)	—
Astara	*Aedes vexans*	—	—	—	—	25 (71.43)	25 (22.7)	32 (76.2)	—
Total	—	—	—	—	10 (5.1)	35 (17.8)	110 (55.8)	42 (21.3)	—

^a^No sample was collected.

### Dominance and Distribution of the *Aedes* Species

*Ae vexans* was an eudominant species (D=75.7%), with a constant distribution (C=100%) in both larvae and adult forms. *Ae geniculatus* showed eudominance and a frequent distribution of up to D=21.2% and C=66.7%, respectively, compared with other species. *Ae echinus* was subdominant (D=2.9) and had a sporadic distribution of up to C=11.1%. Because *Ae pulchritarsis* specimens were collected at low frequencies, its distribution and dominance structures are not discussed (Table 4).

**Table 4:** The dominance and distribution values of larvae and adults of *Aedes* species collected at higher-priority entry points of Guilan Province, Northern Iran from May 2018 to December 2019.

			N (%)		Dominance (%)			Dominance criteria		Distribution (%)		Distribution criteria
**Adults**												
	*Aedes vexans*	367				75.7	Eudominant		100		Constant	
	*Aedes geniculatus*	103				21.2	Eudominant		66.7		Frequent	
	*Aedes echinus*	14				2.9	Subdominant		11.1		Sporadic	
	*Aedes pulchritarsis*	1				0.2	Subrecedent		11.1		Sporadic	
**Larvae**												
	*Aedes vexans*	197		100			Eudominant		100		Constant	

### Biodiversity in Spatial and Temporal Scales

The biodiversity indices of *Aedes* species at spatial and temporal scales are shown in Tables 5 and 6. The Shannon-Wiener Index was calculated to be up to 0.84 and 1.04 in Rasht and July, respectively. Maximum richness (*S*) was found in Rasht (*S*=4) and in all months except in August and November. Menhinick (*D_Mg_*) and Margalef (*D_Mn_*), as indices of species richness, had the highest numerical values in Rasht (*D_Mg_*=0.27; *D_Mn_*=0.56) and July (*D_Mg_*=1.06; *D_Mn_*=0.96). The highest values of evenness (*J*′) were recorded in Anzali (*J*′=0.76) and in July (*J*=0.94). The maximum Simpson diversity index was found in Anzali (*D*=0.74) and jointly in October and November (*D*=0.80), indicating the strong influence of the eudominant species, *Ae vexans*, on other species in the area.

**Table 5:** Biodiversity indices of *Aedes* species in Guilan Province, Northern Iran, by spatial scale, 2018 to 2019.

Species	Rasht	Anzali	Astara
Richness (*S*)	4	2	1
Abundance (*N*)	205	301	176
Menhinick (*D*_Mg_)	0.27	0.11	0.07
Margalef (*D*_Mn_)	0.56	0.17	0
Shannon-Weiner (*H*)	0.84	0.42	—^a^
Simpson (*D*)	0.49	0.74	—
Evenness (*J*)	0.58	0.76	—

^a^Not computable.

**Table 6:** Biodiversity indices of *Aedes* species in Guilan Province, Northern Iran, by temporal scale, 2018 to 2019.

Species	May	June	July	August	September	October	November	December
Richness (*S*)	3	3	3	1	3	3	2	3
Abundance (*N*)	113	39	8	10	59	253	150	50
Menhinick (*D*_Mg_)	0.28	0.48	1.06	0.31	0.39	0.18	0.16	0.42
Margalef (*D*_Mn_)	0.42	0.54	0.96	0	0.49	0.36	0.19	0.51
Shannon-Weiner (*H*)	0.73	0.85	1.04	—^a^	0.50	0.35	0.33	0.73
Simpson (*D*)	0.57	0.45	0.37	—	0.73	0.80	0.80	0.75
Evenness (*J*)	0.69	0.78	0.94	—	0.55	0.47	0.70	0.52

^a^Not computable.

### Rarefaction Analysis

**Figure 4:**
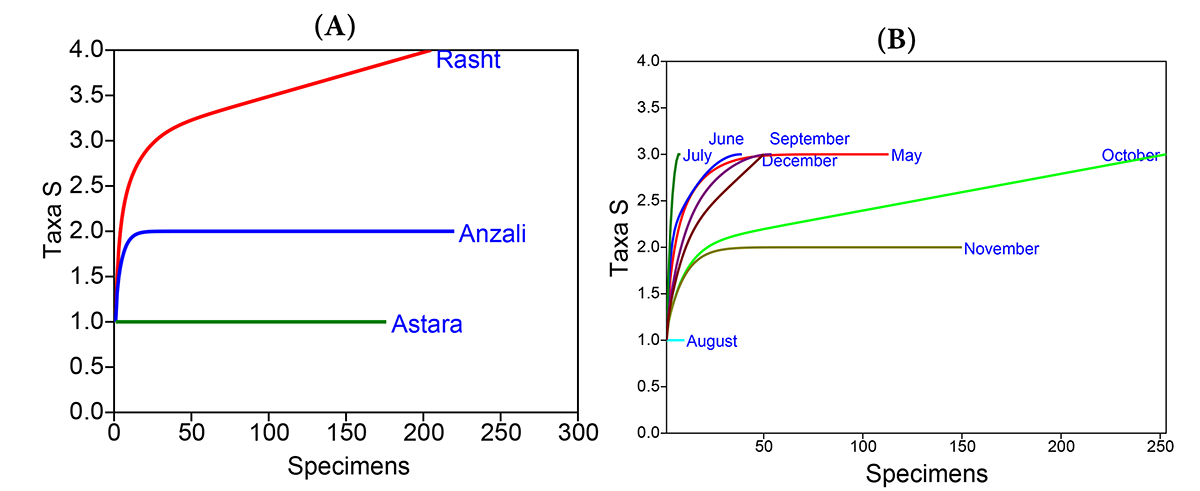
Refraction curve at 95% CI, based on species richness at spatial (A) and temporal (B) scales, 2018 to 2019. Taxa S refers to species richness or number of species.

The rarefaction curves showed the stability of the number of species in each sample (the horizontal axis shows the number of individuals and the vertical axis shows the number of expected species yielded from the method). It almost reached the asymptotic line at all spatial and temporal scales, except in Rasht and in October, where more sampling efforts were needed to increase the richness (Figure 4).

### Effects of Meteorological Factors on the Population of the *Aedes* Species

A significant positive correlation was observed between the population of *Ae vexans* and mean rainfall (*r*=0.521; *P*=.009) and humidity (*r*=0.510; *P*=.011). The mean temperature had a significant negative effect on the *Ae vexans* population (*r*=−0.443; *P*=.035). In addition, no significant relationship was observed between the population of other *Aedes* species and meteorological factors (Table 7).

**Figure 5:**
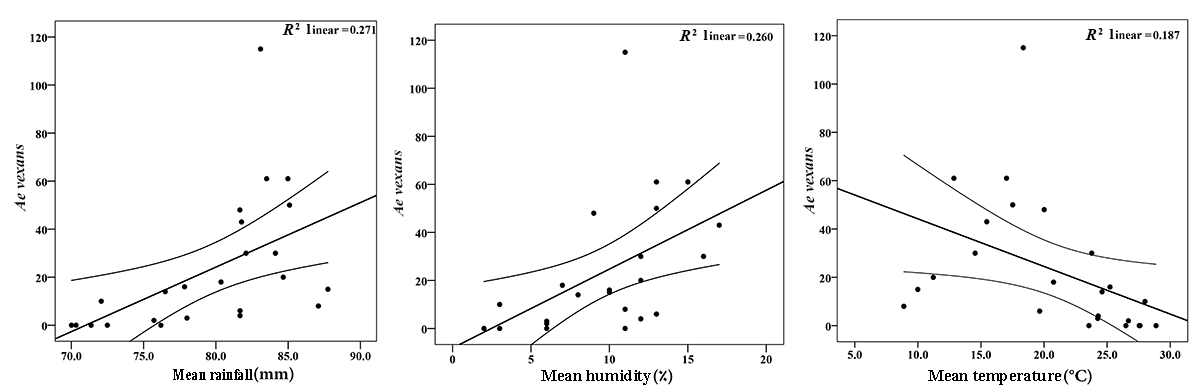
Regression relationships between *Aedes vexans* and meteorological factors at higher-priority entry points of Guilan Province, Northern Iran, from May 2018 to December 2019.

The tested regression model described low *R*^2^ values of 0.27, 0.26, and 0.18 between the *Ae vexans* population and mean rainfall, humidity, and temperature, respectively (Figure 5).

**Figure 6:**
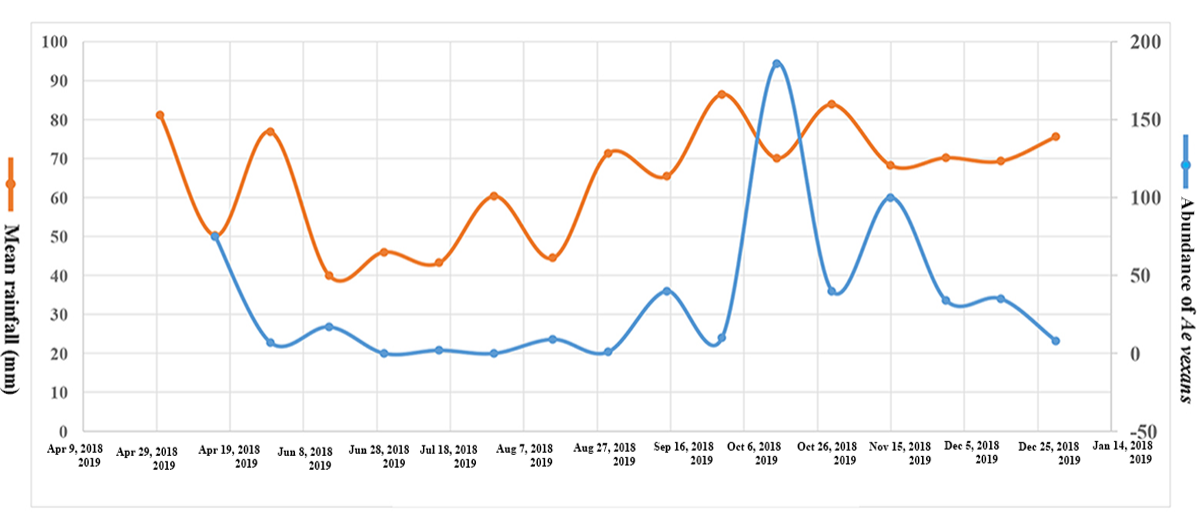
Lag phase between rainfall and population frequency of *Aedes vexans*, Guilan Province, Northern Iran.

Figure 6 shows that after rainfall, with a lag time of approximately 15 days, the *Ae vexans* population increases significantly.

**Table 7:** Correlation coefficient between *Aedes* species population and meteorological factors at higher-priority entry points of Guilan Province, Northern Iran, from May 2018 to December 2019.

Species			Mean temperature (°C)		Mean humidity (mm)		Mean rainfall (%)			
*Aedes vexans*										
	Coefficient	−0.432		0.510		0.521				
	*P* value	.04		.01		.009				
	N	24		24		24				
*Aedes geniculatus*										
	Coefficient	−0.138		0.170		0.272				
	*P* value	.52		.43		.20				
	N	24		24		24				
*Aedes echinus*										
	Coefficient	0.073		−0.170		0.036				
	*P* value	.74		.43		.87				
	N	24		24		24				
*Aedes pulchritarsis*										
	Coefficient	−0.111		0.172		0.217				
	*P* value	.61		.42		.31				
	N	24		24		24				

## Discussion

### Species Composition, Dominance, and Distribution

The introduction, establishment, and spread of invasive *Aedes* species are of great public health concern, mostly because of their ability to transmit a variety of arboviruses [21]. Surveillance is important to detect the occurrence and establishment of uncommon or invasive species, evaluate the risk of pathogen transmission, plan vector control programs, and understand the ecology of circulating vectors and the diseases they transmit in the region [22]. This is the first comprehensive surveillance of *Aedes* species focusing on *Ae aegypti* and *Ae albopictus* at higher-priority entry points in Guilan Province, Northern Iran, performed according to the Iran Centers for Disease Control and Prevention surveillance guidelines for invasive *Aedes* vectors [9]. In total, 3 genera and 13 species of mosquitoes were collected in this study. Furthermore, 5 species belonging to the genus *Anopheles*, 4 species of the genus *Culex*, and 4 species of the genus *Aedes* were collected from the study areas. Although species other than *Aedes* are also important vectors of some pathogens, only the *Aedes* species are discussed here.

*Ae aegypti* and *Ae albopictus* were not found in the sampling efforts at the points of entry and high-risk sites in this study. It is worth mentioning that historically, *Ae aegypti* was observed in the Khuzistan and Bushehr provinces of southern Iran [23-25]. However, this species was not observed in Iran from 1954 to 2019. Malaria eradication programs in Iran, which began in 1957, could be a reason for its disappearance. Recently, *Ae aegypti* has been detected in the ports of Khamir and Lengeh, as well as Bandar Abbas in Hormozgān Province [9], which makes its re-establishment plausible and poses a potential risk of its spread to other parts of the country.

The Asian tiger mosquito, *Ae albopictus*, was found for the first time in Iran in the districts of Nik-shahr, Sarbaz, and Chabahar in Sistan and Baluchestan Province [12]. It has been the most invasive mosquito species worldwide over the past 30 years [6]. This thermophilic species is adapted to a more temperate climate by producing diapausing eggs and has a strong tendency to expand, as the species was observed in 28 European countries, established itself in 20 of them [26,27]. It should be noted that extensive entomological surveillance has failed to reproduce observations of *Ae albopictus* after its first presence in Iran. This reflects the fact that *Ae albopictus* may not have been able to establish itself in the area [28].

*Ae vexans* was the eudominant species with a constant distribution in both the larval and adult stages. The species was classified as a dominant species with varying distributions, including sporadic [17], moderate [29], and infrequent [30]. In comparison with our research, *Ae vexans* was introduced as a satellite species with a sporadic distribution [31]. It is widely distributed throughout the Holarctic region and is native to Eastern Europe, the Americas, Africa, and Asia [32,33]. It was introduced as the most prevalent species of *Aedes* in Northern Iran [17,34], which is in accordance with the findings of this study.

The floodwater mosquito *Ae vexans* is an opportunistic feeder that can feed on birds and mammals, facilitating zoonotic transmission [35]. Apart from being known as a biting pest, the species is known to be a competent vector for St Louis encephalitis virus, Snowshoe hare virus, Jamestone Canyon virus, Tahyna virus, Batai virus, and the dog heartworm parasite *Dirofilaria immitis* [36]. *Ae vexans* is considered the primary vector of Rift Valley fever phlebovirus in Africa [37], and its transmissibility has been experimentally confirmed in field populations of the Americas continent [38]. Zika virus has recently been detected in the salivary glands of *Ae vexans* caught in the field [39]. *Ae vexans* is assessed as a potential secondary vector of West Nile virus [35] and has been considered as a main “bridge vector” owing to its preferred blood-feeding habits of both humans and birds. Owing to the high abundance of the species, the potential role in virus transmission, and the existence of wetlands for migratory birds as the reservoir hosts in the study area, the ecological aspects of *Ae vexans* could be the focus of future research.

*Ae geniculatus* was recorded in the dominant class with a frequent distribution. The species has been reported to be dominant in the forest habitats of the northern regions of Iran (without reporting any dominance and distribution indices) [40,41]. Similar to invasive *Aedes*, this species is known as the tree trunk hole mosquito, breeds in natural containers in woodlands and in man-made containers in semiurban and semidomestic environments [42]. *Ae geniculatus* is a Palearctic species, opportunistic feeder, competent vector for *Dirofilaria immitis* and *repens* [42,43], and chikungunya [44]. The species was not collected during sampling efforts from Astara County in this study, which may be because of the lack of preferred habitats and differences in selected sampling sites during the monitoring period. Because there is little data regarding the biology, ecology, and pathogens transmitted by the species in Iran, further research is needed to increase the knowledge on these issues in the future.

*Ae echinus* was a subdominant species with an infrequent distribution and was found only in Rasht County in our investigation. The differences in the sites selected during the study were probably a limiting factor. The species is distributed in the Mediterranean region, North Africa, Asia and Southern Europe, Greece, Algeria, Morocco, Spain, and France [45]. *Ae echinus* specimens were collected for the first time in Sari, Mazandaran Province by Janbaksh in 1955 [46], and in the counties of Rezvanshahr, Shaft, Fuman, and Masal of Guilan Province by Azari-Hamidian in 2002 [47] as larvae. By contrast, in this study and another study in Northern Iran [41], this species was observed only in the adult form.

*Ae pulchritarsis* specimens were collected at a low frequency. All *Aedes* species collected in the study are native to the northern regions of the country, as shown in the checklist of mosquitoes in Northern Iran [17,48].

### Monthly Population Trends of the *Aedes* Species

Climate can accelerate or delay the development of mosquitoes and the availability of breeding sites [49]. An overall trend of a lower number of *Aedes* mosquitoes being observed in the drier months (June to September) than during the wetter months (October to May) was evident (Figure 2). The results of this study showed that the most prevalent species, *Ae vexans* and *Ae geniculatus,* were mostly observed in the autumn, when it can be the most appropriate starting to start monitoring the population dynamics of these species. This finding was further supported by Wagner and Mathis [50]. The population fluctuations of these species began in May, peaked in October, and gradually disappeared after December. There is not much data about the seasonal activity of *Aedes* species in Iran. The maximum population densities of *Ae geniculatus* and *Ae vexans* were reported in September and October, respectively, in Mazandaran Province, Northern Iran [41,51]. The highest activity peaks of *Ae vexans* were documented in June [29] and August [52] in the Iğdır Plain of the Aras Valley, Turkey. The difference between the results of this study and the findings in other regions may be explained by the topography, climate, and sampling sites selected, which affect the bionomics of *Aedes* mosquitoes.

### Effects of Meteorological Factors on the Population of the *Aedes* Species

*Ae vexans* population showed a significant positive correlation with mean rainfall and humidity and a negative correlation with mean temperature. In concordance with our findings, many studies have shown the effects of meteorological factors on the *Aedes* population dynamics [51,53-56]. The researchers believed that rainfall was the most influential climatic variable for the *Ae* *vexans* population, sometimes with a lag phase, by creating temporary pools as the preferred habitat for floodwater species. This study was not designed to and did not aim to address the analysis of lag time, but according to Figure 6, it seems that there is probably a lag time of at least 15 days from the beginning of the rainfall to the observation of an increase in the population of *Ae vexans*. In agreement with our findings, there was a lag time of at least 10 and 15 days in the early rainy season and 20 days after the end of the rainy season between the peak of rainfall and abundance of the species. Some studies have also shown that there is a complex relationship between rainfall and the *Ae vexans* population trend; in 2005, rainfall had a negative

effect on the population density of *Ae vexans*, and in 2006, it had a positive effect [57]. These findings suggest that seasonal activities should be evaluated over several years to gain a better understanding of the lag time between population trends and climatic factors as well as the impact of other variables.

Temperature can be considered a survival-limiting factor for populations of the *Aedes* species [56]; this is supported by this study. Relative humidity was positively and significantly correlated with the population dynamics of *Ae vexans* [55], which is in agreement with our investigation. However, there was no positive or negative correlation between the abundance of other *Aedes* species and meteorological factors.

### Biodiversity and Rarefactions Analysis

There were differences in the biodiversity of *Aedes* species at the spatial and temporal scales in the study area, as shown by the maximum values of the Shannon-Weiner index in Rasht and in July. Suitable habitat conditions for mosquitoes to reproduce and thrive could be a reason for this high diversity. Some studies have shown that lower biodiversity in a community might lead to a faster rate of emergence and re-emergence of infectious diseases [58-60]. They believed that there was a link between high biodiversity and reduced risk of vector-borne diseases, which underscores the importance of biodiversity studies. This is in agreement with the opinions of other researchers.

The highest levels of richness were observed in Rasht; these values were the same in all months except in August and November. Given that species richness is affected by sampling intensity, a standard rarefaction curve was used to confirm the adequacy of sampling efforts at temporal and spatial scales by reaching the asymptotic line. Rarefaction curve analysis showed that further sampling efforts are required to achieve maximum richness in Rasht and in October.

Evenness is presented as how individuals are distributed in a community, with the highest rates observed in Anzali and in July. Although there was high evenness in Anzali, the greatest diversity was observed in Rasht. This is because the biodiversity index is influenced by 2 other factors, that is, species richness and dominance. Low or high rates of these factors can affect the biodiversity index [18,61]. There are no specific data related to the biodiversity of *Aedes* species in Iran, and only a few studies have sporadically measured the biodiversity indices of mosquitoes in Neka city, Mazandaran Province, by Nikookar et. al [18]; in Bashagard district, southern Iran by Hanafi-Bojd et al [62]; in Abhar County, Azerbaijan Province by Paksa et al [63]; and in Chaharmahal and Bakhtiari Province by Omrani and Azari-Hamidian [64], highlighting the need for more studies in this regard.

As there is no proper vaccine available for *Aedes*-borne diseases (dengue, Zika fever, and chikungunya) to date, and because of the relatively high insecticide resistance in *Aedes* vectors, the main control intervention would be source reduction. Therefore, community participation is a key important factor that has shown varying degrees of success in disease prevention [65]. Raising people’s awareness through social media, holding educational workshops [66,67], and using mobile phone–based monitoring apps to produce risk maps [68] can be useful in community-based vector control.

### Conclusions

According to the findings of this study, although Guilan Province has the potential for hosting the invasive vectors, *Ae aegypti* and *Ae albopictus*, these species were not found in this region during the monitoring period. However, entomological surveillance of *Aedes* mosquito fauna is important at entry points and high-risk sites for the timely identification of the entry of invasive species. *Ae vexans*, which is a potential vector of medical and veterinary importance, actively circulates in autumn in Guilan Province, Northern Iran. Other important *Aedes* species have also been identified in the study areas. Our data can be useful to health policy makers in designing and implementing appropriate surveillance and control measures for *Aedes* mosquitoes.

## Appendix

Multimedia Appendix 1 Ecogeographical characteristics of sampling sites where mosquitoes were collected in Guilan Province, Northern Iran.
