# Supplementary material for: Entomological Surveillance of the Invasive Aedes Species at Higher-Priority Entry Points in Northern Iran: Exploratory Report on a Field Study
Source: JMIR Public Health Surveill. 2022 Oct 31;8(10):e38647. doi: 10.2196/38647 (PMC9664326; doi:10.2196/38647)
Supplement: Multimedia Appendix 1 [file publichealth_v8i10e38647_app1.docx]

Appendix 1. Eco-geographical characteristics of sampling sites where mosquitoes were collected, in Guilan Province, northern Iran.

| **Township** | **Stations** | **Longitude (E)** | **Latitude(N)** | **Altitude (m)** | **Description (**Building structure, vegetation cover, Water shed**)** |
| --- | --- | --- | --- | --- | --- |
|  | Saravan village | 49°39′40″ | 37°03′49″ | 72 | The station had adjoining houses with gabled and wooden roofs and concrete and mud walls, covered by dense and tall trees, meadows, shrubs, flowers with natural larval habitat (tree trunk holes, wetland, forest pits), man-made larval habitat (buckets, tires, water collection channels, pits) and water storage tanks |
| Rasht | Sardar Jangal Intrnational Airport | 49°36′59″ | 37°19′15″ | -12 | The airport had only a large office building for staff and a waiting room for travelers. The building was made of iron and concrete with gabled roofs. The area was covered with grass and flowers and small shrubs with natural larval habitats (water collected under shrubs, grasses during irrigation and or rainfall, etc.) and artificial (buckets, rainwater outlets, water dripping from coolers, etc.). |
|  | Balakoikh village | 49°36′53″ | 37°19′11″ | -13 | The houses at this station were at a distance of 1-50 meters from each other with gabled roofs and concrete and mud walls, located in a close vicinity to the airport, covered by vegetation and trees, pastures, shrubs, flowers with natural and artificial larval habitat (tree trunk holes, Wetlands, forest pits, buckets, tires, pots, pits) and water storage tanks. |
| Anzali | Ports and Maritime Administrations | 49°28′19″ | 37°28′32″ | -23 | The station had an office building, a department store, villas for employees to rest on, at a distance of approximately 3-50 meters from each other, with gabled roofs and concrete walls, covered by tall and short trees, shrubs, flowers, grass with natural and artificial larval habitat. There was a large wetland covered with reeds near the station. |
|  | Gomrok | 49°28′15″ | 37°28′28″ | -23 | The station had small and large office buildings with gabled roofs and concrete-iron structures, at a distance of approximately 5-100 meters from each other, covered with tall and short trees at a great distance, shrubs, flowers, grass with natural and artificial larval habitat. There was a large wetland covered with reeds near the station. |
|  | Caspian tourism area | 49°40′01″ | 37°27′21″ | -28 | Large commercial buildings with a variety of large and varied stores and restaurants, with roofs and concrete-iron structures at close distances, covered with shrubs and green spaces with natural and artificial larvae habitat. |
| Astara | Gomrok | 48°52′42″ | 38°26′08″ | -27 | The buildings had concrete-iron structures with gabled roofs , covered by sparse small trees, shrubs and green spaces with natural and artificial larvae habitat. |
|  | Harbor | 48°52′30″ | 38°24′17″ | 19 | The buildings had concrete-iron structures with gabled roofs, sparse small trees, shrubs and green spaces with natural and artificial larvae habitat. |
|  | Hudul village | 48°49′10″ | 38°24′10″ | 12 | The buildings had gabled and wooden roofs with concrete and mud walls, sparse small trees, pastures, shrubs, flowers with natural and artificial larvae habitat. |
